# Electrochemical Flow Reactors: Mass Transport, iR Drop, and Membrane-Free Performance with In-Line Analysis

**DOI:** 10.1021/acselectrochem.4c00167

**Published:** 2025-01-14

**Authors:** W. J. Niels Klement, Elia Savino, Sarah Rooijmans, Patty P. M. F. A. Mulder, N. Scott Lynn, Wesley R. Browne, Elisabeth Verpoorte

**Affiliations:** † Molecular Inorganic Chemistry, Stratingh Institute for Chemistry, Faculty of Science and Engineering, University of Groningen, Nijenborgh 3, 9474AG Groningen, The Netherlands; ‡ Pharmaceutical Analysis, Groningen Research Institute of Pharmacy, University of Groningen, Antonius Deusinglaan 1, 9713AV Groningen, The Netherlands; ¶ Institute of Physics of the Czech Academy of Sciences, Na Slovance 1999/2, 18200 Prague, Czechia; § Pharmaceutical Analysis, Groningen Research Institute of Pharmacy, University of Groningen, Antonius Deusinglaan 1, 9700 AD Groningen, The Netherlands

**Keywords:** Electrochemistry, Flow, Raman spectroscopy, In-line analysis, Mixing, Microfluidics

## Abstract

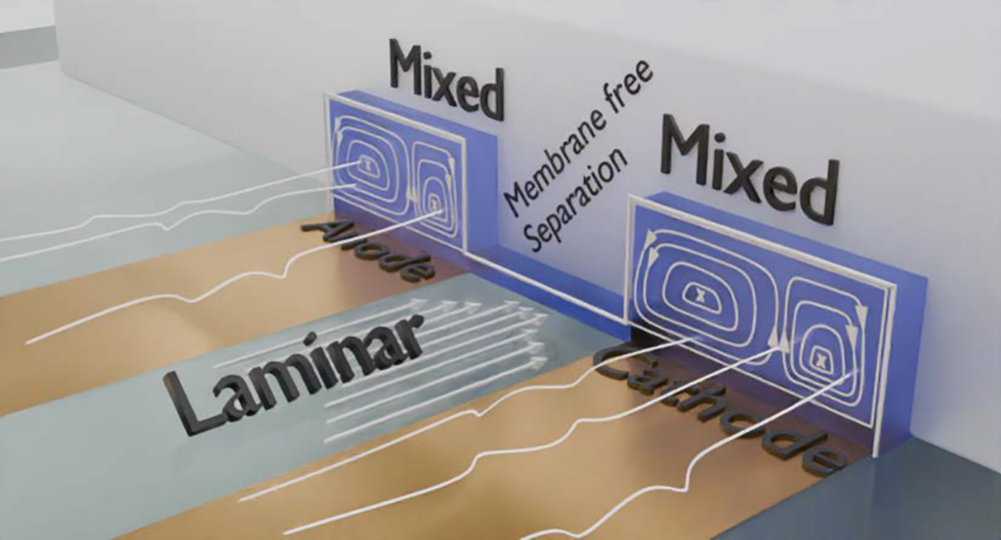

Continuous flow reactors are promising for electrochemical conversions, in large part due to the potentially rapid refreshment of reagents over the electrode surface. Microfluidic reactors enable a high degree of control over the fluid flow. Diffusion to and from the electrode and electrode area determine the efficiency of electrochemical conversion. The effective electrode area is limited by the loss in electrode potential due to iR drop, and further electrode length (and hence area) is limited due to ineffective mass transport to and from the electrode. Here, we report on a microfluidic electrochemical device with large (long) area electrodes running in parallel, which both minimizes the iR drop and ensures a constant electrode potential along the whole length of the electrodes. The electrodes are separated by laminar flow in the channels, instead of by a membrane, thereby reducing cell resistance. Herringbone grooves are used to increase mass transport rates by inducing transverse flow. We confirm fluid flow behavior in the devices using computational fluid dynamics (CFD) and verify the results experimentally using in-line and off-line UV/vis absorption and resonance Raman spectroscopy. We anticipate that this approach will aid future development of electrochemical flow reactors, enabling larger area-electrodes and realizing greater efficiencies.

## Introduction

Electrochemical reactions are emerging as a standard method in fine chemical conversions.^1−6^ The increasing availability of (green) electricity makes electrochemical conversions attractive for the synthesis of fine chemicals^7^ and pharmaceuticals^5^ as well as analysis.^8−10^ The range of classes of reactions that can be driven electrochemically is increasing rapidly.^6^ Furthermore, the electrochemical production of fine chemicals, especially at the point of use, for example, active pharmaceutical intermediates, is a critical step in realizing more sustainable chemical synthesis.^3,5,11,12^

Electrochemical conversions carried out in flow provide advantages in possibilities towards scale up, mass transport, and heat exchange.^1,13^ In particular, microfluidic flow cells provide excellent control over the fluid flow. Such control is especially important in synthetic reactions, e.g., with radioisotopes that need to be carried out rapidly,^5^ or for sampling in analytical chemistry.^8−10^ Additionally, continuous flow operation can ensure a constant delivery of fresh solute to the electrode and removal of products after, which limits further electrochemical steps.

**Figure 1:**
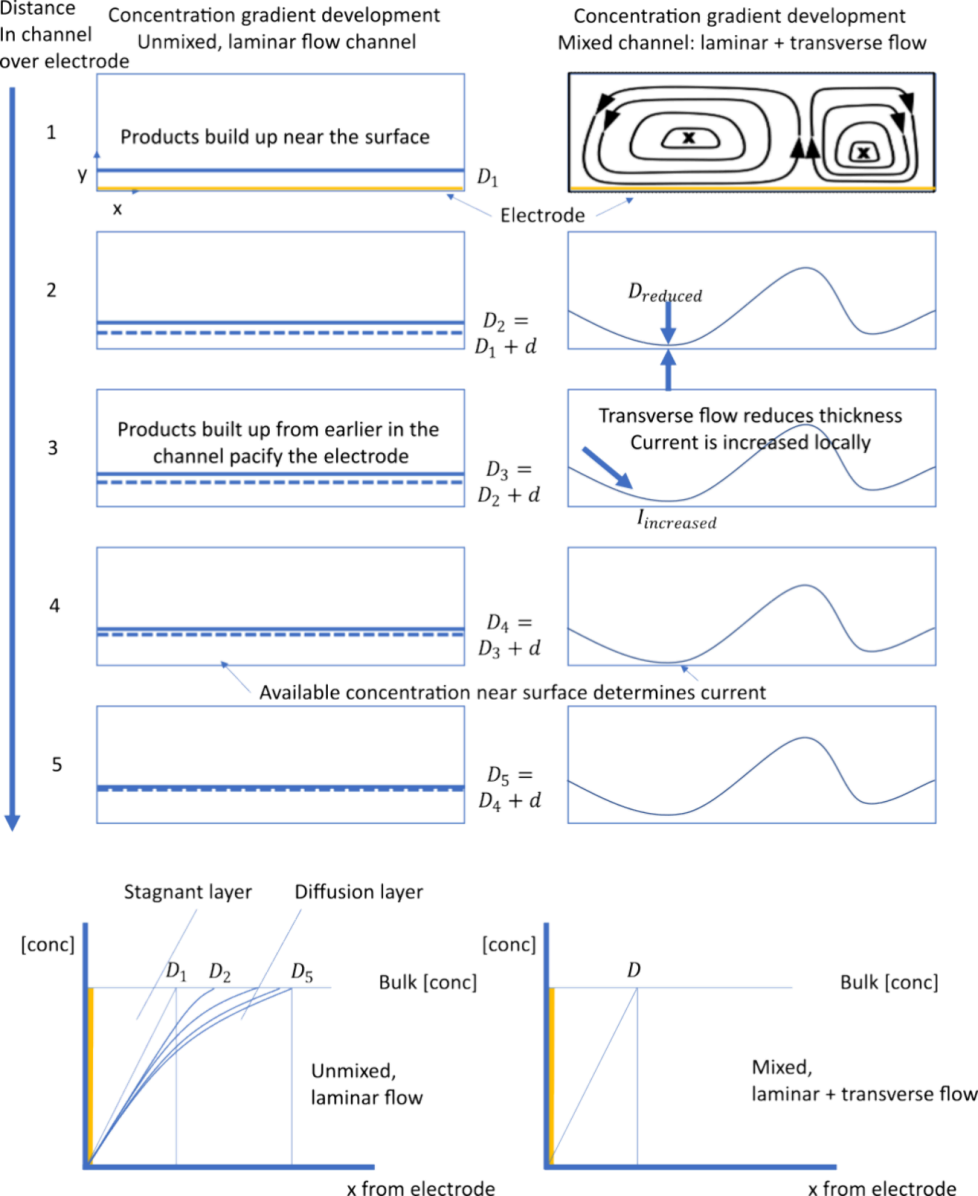
Concentration gradient above the electrode surface determines the achievable current. When a potential is applied, reagents react near the surface: a diffusion layer (*D*) develops (+*d*). Under laminar flow conditions (left), this diffusion layer increases in thickness along the length of the electrode (*D*_2_ > *D*_1_, etc.). A thicker diffusion layer reduces the efficiency of electrolysis and thus efficient use of the electrode surface area further down the channel. In mixed flow conditions (right), transverse flow serves to reduce the thickness of the diffusion layer, which increases the availability of reagents at the electrode surface. Therefore, electrodes in a mixed flow configuration are less affected by the buildup of a diffusion layer and may have an increased achievable current.

Mass transport is a key parameter in electrochemistry since redox active species need to be at (within nanometers) the electrode to undergo electron transfer. A volume depleted of reagent forms at the surface of an electrode when a potential is applied, which is called the (Nernst) diffusion layer.^14^ The diffusion layer thickness is determined by mass transport (diffusion): the reagent has to reach the electrode to react, which limits the overall electrochemical efficiency. This limitation is especially important under flow, where the thickness of the diffusion layer (normal to the electrode surface) increases along the length of the electrode in the direction of flow (Figure 1). Under ideal axial laminar flow conditions, the buildup of the diffusion layer results in mass transport limitations. The increasing distance for reagent diffusion along the channels limits/reduces current and, thus, conversion, and hence, these limitations are particularly important in microfluidic electrochemical cells.^1,2,15,16^

The diffusion layer (D), depleted of reagent, is established quickly in the volume near the surface of the electrode once a sufficient overpotential is applied. Its thickness increases along the length of the channel (Figure 1 left).^1^ Increasing axial flow rates will thin the layer; however, this comes at the cost of a decrease in residence time of reagents in the channel, reducing the extent of conversion achieved. An alternative approach is to thin the diffusion layer using a transverse flow induced by static mixers. However, such mixing may cause undesirable exchange of material between the anode and cathode (Figure 1 right).

**Figure 2:**
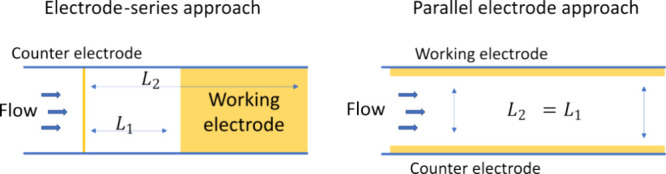
Separation between the working and counter electrode determines the actual electrode potential at a given location due to the distance dependence of electrolyte resistance and hence the voltage loss due to iR drop. In a configuration where the two electrodes are placed at different positions along a microfluidic channel (electrode-series, left), the potential at the working electrode decreases along its length. In a parallel arrangement (parallel electrodes, right), the separation between the working and counter electrode is constant over the entire length, which provides a constant electrode potential.

An approach taken to separate the anode and cathode in flow reactors is to direct the flow over the counter electrode first to avoid decomposing the products formed at the working electrode.^8^ The electrodes are placed in series so that products from the second electrode do not contact the first. This arrangement is effective in separating the electrodes but increases the impact of iR drop (the loss in potential due to solution resistance). The difference in electrode separation (*L*) introduces a gradient in the working electrode potential. The distance between a point on the surface of the working electrode and the counter electrode is proportional to an increase in iR drop, which limits the effective electrode area, Figure 2. Essentially, the in-series arrangement of electrodes limits the maximum electrode area significantly and hence the conversion that can be achieved.

Indeed, when a high extent of conversion is desired, large, effective electrode areas are needed.^1,16^ This can be achieved, for example, with large parallel plates with small inter-electrode gap and thin channels in between the electrodes.^1,2,13,16^ Such devices are effective for many reactions, but for others, separation of anode and cathode solutions (a divided cell) can be essential.^6^ Therefore, aligning the anode and cathode in parallel along a channel may require ion conductive membranes or large electrode–electrode separations to reduce mass transport and, hence, mixing of solutions between the two electrodes.^2,15,16^ Membranes are effective in separating electrode compartments, but for fast prototyping fabrication, devices without membranes can be advantageous. An alternative approach to obtaining separation is through the use of laminar flow, as described by Horii et al.,^17^ which is effective in separating the anolytes and catholytes. However, this approach is not compatible with the introduction of the static mixing structures needed to induce transfer flow in the channels needed to increase the mass transport efficiency.

An alternative approach explored in fuel cell devices is to arrange electrodes in parallel with a small inter-electrode gap to reduce iR drop and not use a membrane.^12,18^ Laminar flow is used in such devices to separate the electrode compartments, instead of a membrane. The absence of a membrane simplifies fabrication and significantly reduces resistance, thus increasing the electrical efficiency and improving the currents that can be achieved in the fuel cells substantially.^12,19^ In microfluidic redox flow batteries, maximum currents can be maintained by using high flow rates to refresh reagent at the electrode surface efficiently and sustain faradaic currents. Marschewski et al. have shown that introducing herringbone static mixing structures enhances mass transport in membrane-less microfluidic cells for redox flow batteries.^20^

The membrane-free approach used in fuel cells and redox flow batteries presents a challenge in cells focused on chemical conversions and mass efficient electrolytic operation: mass transport. Continuous flow delivers fresh reagents to the electrode surface. However, this process does not provide for a significant increase in the extent of redox conversions, since the diffusion layer develops over the electrode surface as a result of the axial laminar flow that is present due to the small characteristic sizes of microfluidic devices.

**Figure 3:**
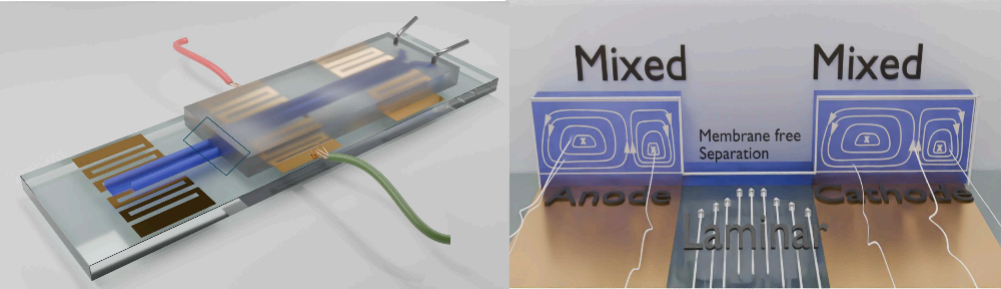
Design of the reported microchannel. The chip features two channels, one for the anode and one for the cathode, with long parallel electrodes yielding large effective electrode surface areas. The solutions flowing through the channels are mixed using herringbone static mixers, which bring fresh solute to the electrode surface by transverse flow. The anode and cathode are connected by an open bridging channel. Laminar flow in the bridge prevents transverse migration of the solute between the anode and cathode channels. The laminar regime arises in between the two main channels, as they are connected by a 10 micrometer high bridge throughout their length. The bridging section is not directly subjected to flow by the pumps and is not mixed by the static mixers and is therefore relatively undisturbed.

**Figure 4:**
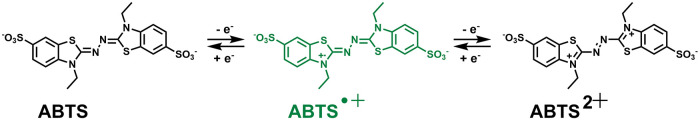
Structures of ABTS and its one- and two-electron oxidation products, from left to right. The one electron oxidation product, ABTS^+•^, is colored green.

In the present study, we demonstrate a membrane-free electrochemical flow cell, where the anode and cathode cells are divided by laminar flow instead of a membrane, Figure 3. The anode and cathode are long parallel electrodes covering a significant length of the device to reduce the iR drop and maintain a constant potential over the entire electrode area. Additionally, we show that improvement in efficiency of these cells for electrochemical conversions is achieved using static herringbone mixers. The static mixers included in the electrode-containing part of the channels substantially increases the flux of reactants to the electrode surface^9,10,20^ by thinning of the diffusion layer on the electrode surface through the transverse flow they generate. In principle, the herringbone design is optimized for mixing of fluids rather than delivery of solute to the electrode surface. However, the key parameter to mix fluids (induced transverse flow) is also essential for the delivery of fresh solute to the electrode surface.^9,10,20^ The improved productivity, channel, and flow properties are characterized and visualized using computational fluid dynamics (CFD) and analyzed spectroscopically in-line and off-line using ABTS (2,2′-azino-bis (3-ethylbenzothiazoline-6-sulfonic acid)), Figure 4, a common redox probe used in (bio)chemistry.^21,22^ ABTS is chosen because its one-electron oxidation yields a green color, observable both by eye and by resonance Raman spectroscopy due to its broad absorption around 700 nm.

The experimental data are compared with simulated results for the analysis of the current design. We show that static mixers reduce and change the shape of the diffusion layer through the transverse flow they induce.^10^ Reducing the diffusion layer thickness significantly improves the reaction efficiency and rate by enhancing mass transport, crucial for optimizing device performance. Essentially, it helps to bring fresh solute to the electrode and carry the product away from the electrode. This optimized performance is shown by the generation of hydrogen peroxide at the platinum electrodes.

## Results and Discussion

An essential feature in the design of the electrochemical microfluidic cell is the 10 micrometer high bridge structure that supports only axial laminar flow and connects the anode and cathode channels as a single structure. This design for the connection between the channels prevents mass transport of solvent between each main stream (*vide infra*) and ensures that the anode and cathode channels are kept separate.

**Figure 5:**
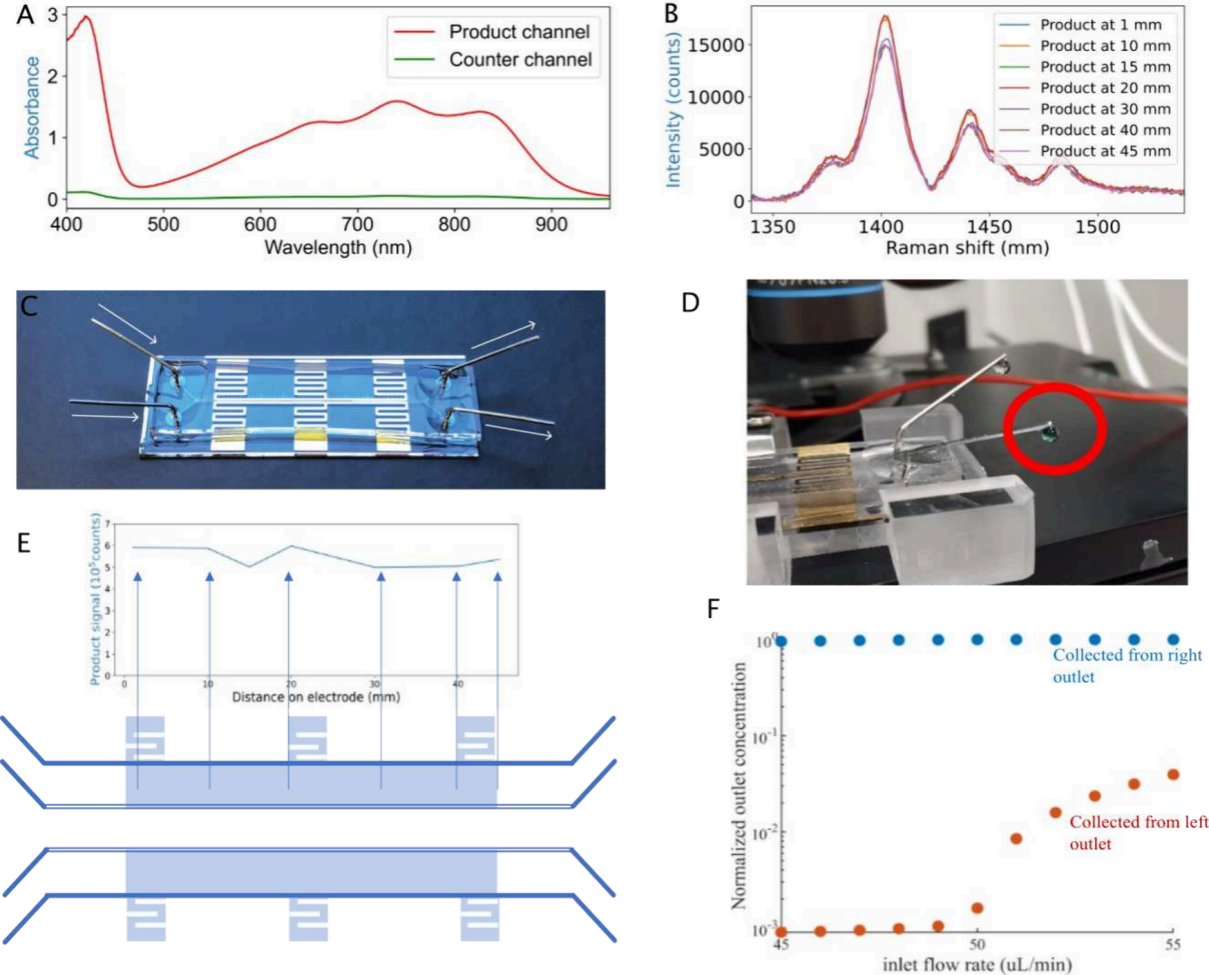
(A) Absorption spectra of contents exiting each of the channels after electrolysis. The colored product ABTS^•+^ is observed only in the channel in which the electrode is polarized positively. (B) Raman spectra of ABTS^•+^ containing electrolyte solution during iR drop experiments. Spectra are recorded at selected locations along the length of the channel that was held briefly at a positive potential, using a 1 V potential difference across the channels. The spectral intensity is similar at all points, indicating equivalent extents of oxidation along the electrode’s length. (C) Photograph of the chip. The base of the chip containing the electrodes and connection pads for the potentiostat features a zig-zag pattern. The zig-zag connects the pads to the electrodes with minimal reduction of bond strength between the top and bottom parts of the chip. Polydimethylsiloxane (PDMS) can adhere to the glass between the zig-zag lines, which provides extra strength and significantly reduces leaks. (D) Photograph of the outlets, with droplets emerging. In this case, this was under applied potential. The droplet to the right is green in color, indicating ABTS^•+^ oxidation at this electrode. The droplet to the left is not green, indicating the separation of the two main channels. (E) Integrated Raman intensities of products in (B). A relatively constant amount of ABTS^•+^ is observed over the length of the channel, indicating a constant iR drop. (F) Simulation of transverse flow between anode and cathode channels for various flow rates. Transverse flow by diffusion between channels is minimal considering the residence times relevant to the current study (see the text for details).

**Figure 6:**
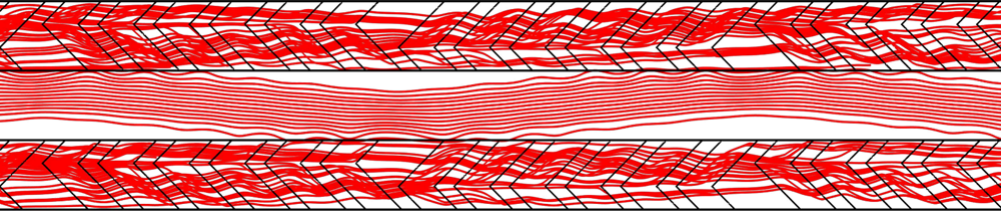
Simulated streamlines in the microfluidic device. Streamlines, flowing left to right, visualize the flow profile of particles entering the start of the channel and flowing through. The separation of channels is apparent here. Specifically, the transverse flow between the top and bottom mixed channels is not observed.

The separation of the solutions flowing in each channel is readily apparent when a colored product is formed electrochemically (Figure 5). For example, the bright green product of ABTS oxidation (i.e., ABTS^•+^) can be observed by the naked eye, Figure 5D, as it emerges from the anode channel only, Figure 5A. The separation of channels can be visualized by simulated streamlines in the channels, Figure 6. An absence of transverse streamlines between the main channels indicates the absence of mass transport between them. The counter electrode, in water, is essentially a standard hydrogen reduction electrode (normal hydrogen electrode, NHE), which allows for potentials to be applied relatively accurately.

The convective transport of species formed at the electrode in one channel to the other channel does not appear to be substantial, even with the transverse flow induced when static mixers (slanted herringbone mixers (SHM)) are present. Furthermore, transport by diffusion across the 300 micron wide bridge separating the electrode-containing channels is limited by the residence time of solutes, ca. 600 ms at 100 μL/min, considering a typical diffusion coefficient for a small molecule (5 × 10^–6^ cm^2^ s^–1^). Residence time is estimated from the electrode channel volume (300 micron wide × 76 micron (average height) × 4.5 cm = 1.03 μL) and the flow rate (100 μL/60 s).

Simulations (CFD) indicate an expected extent of crossover below one percent of the total concentration of analyte over a range of flow rates, Figure 5F and Figure 6.

The behavior anticipated by simulations was confirmed by the minimal extent of crossover of a blue solution, entering one channel of the device, to the other channel, Figures S1, S2, and S3. Hence, the crossover of species formed at the electrodes can be disregarded by the flow rates used here. In principle, transverse migration can be avoided fully by a slight increase in pressure in one of the channels (by increasing flow rate).

### iR Drop and Effective Electrode Area

The large electrode area (13.5 mm^2^, 4.5 cm long × 300 micron wide) in the current chip design is essential to achieve significant extents of oxidation/reduction of substrates flowing through the channels. It is essential that the iR drop between the electrodes remains constant over the entire length of each electrode to avoid that the electrode potential varies, and hence, conversion efficiency is diminished. The parallel arrangement of the electrodes achieves this, with an expected maximum iR drop of ca. 10 mV, i.e., 1% for a 1 V potential difference.

The variation in potential along the electrode in the channel was determined through a stop-flow experiment: the channels were filled with a solution of ABTS, and the flow stopped before a 1 V potential difference (corresponding to approximately the  of the first oxidation of ABTS) was applied for 0.5 s. The extent of oxidation to ABTS^•+^ along the electrode, determined by in-line Raman spectroscopy,^23^ shows that the local electrode potential does not vary significantly, i.e., the extent of conversion to ABTS^•+^ due to the potential pulse being similar at all points, Figure 5E. The intensity of the Raman scattering from ABTS^•+^ along the channel length is dependent on electrode potential at each location. We conclude that the electrode potential is constant over the whole length of the channel.

### Slanted Herringbone Grooves to Increase Solute Flux to the Electrode

**Figure 7:**
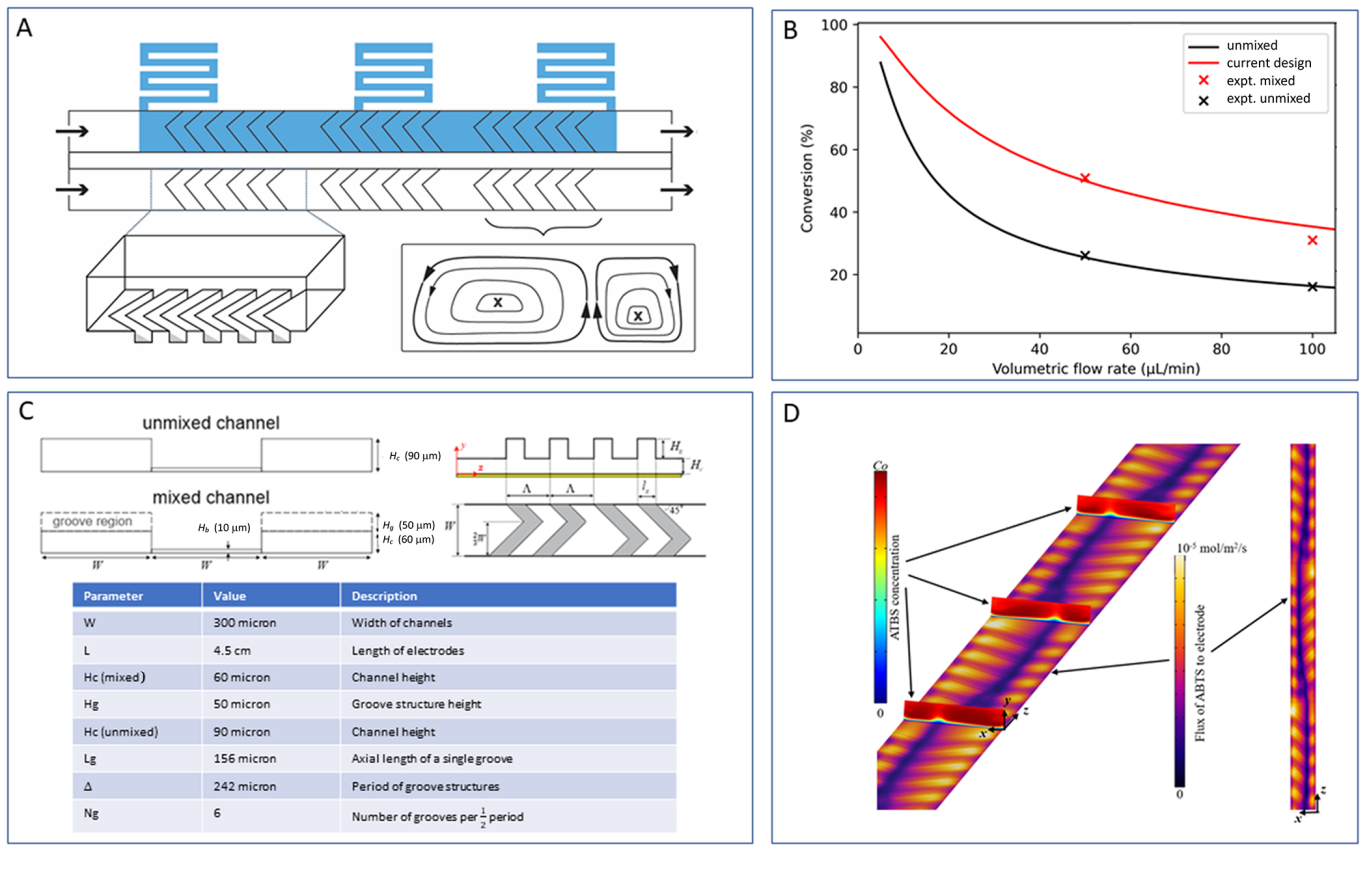
(A) Channel layout showing three half cycles of herringbone grooves in each channel. The grooves are present in the ceiling of the channels, and direct flow up and to the side of the channel. This flow profile creates a double vortex inside the channel, which increases the transverse mass transport. (B) Comparison of analytically calculated and experimentally observed conversion of reagent inside a mixed and unmixed channel. The conversion decreases with an increasing flow rate due to the reduced residence time on the chip. The predicted conversion for the current design of channel including slanted herringbone grooves for static mixing is higher than unmixed channels at all flow rates and matches well with experimental data, Figure 5A. (C) Parameters used in fabrication of the chip and in the computational fluid dynamics simulations. (D) Simulated flux of solute near the electrode surface. (yellow) High flux of fresh solute to the electrode surface (thinning of diffusion layer) and (blue) product swept away from the electrode, forming an upwelling of product in the channel.

The flow rate determines the residence time of solutes in the electrode containing channel. Using lower axial flow rates allows for the buildup of the diffusion layer and, hence, lower current but increases the residence time, allowing for a greater extent of conversion, Figure 7B. In a smooth-walled channel, product accumulates near the electrode interface. The laminar flow profile in the channels limits electrochemical oxidation/reduction as only molecules that can diffuse to the electrode within the residence time in the channel can be converted, forming a boundary layer (diffusion layer). Since diffusion is the sole form of mass transport in the non-axial (transverse) direction, inducing transverse convection allows the boundary layer to thin without reducing residence time in the channel. This is achieved using herringbone grooves inside the microchannel, Figure 7A. Kirtland et al.^24^ reported a theoretical analysis of transport in channels containing such static mixers and demonstrated how the convection expected impacted conversion over a range of flow rates, Figure 7B. The introduction of these structures, with parameters noted in Figure 7C, predicts substantial increases in efficiency (+100%) over a wide range of flow rates (50–100 μL min^–1^). Notably, the difference in conversion between the mixed and unmixed channels is maximized around 50 μL/min, above and below which the difference in conversion will be decreased. This behavior stems from the complex behavior of heterogeneous solute transport in mixed channels: in the limit of low flow, the boundary layer in both mixed and unmixed channels will encompass the entire channel (i.e., conversion will approach 100%), whereas at high flow rates, the boundary layers in both channels will remain thin enough such that mixing has a reduced effect.

Specifically, the additional mass transport increases the flux of the reagent to the electrode (see Figure 7D for computational fluid dynamic simulations). The (Nernst) diffusion layer is locally reduced by convection orthogonal to the direction of flow, similar to that observed with a rotating disc electrode, or earlier works focused on analyte delivery to a surface for sensing applications.^25,26^

The simulated results were verified experimentally with off-line analysis by UV/vis absorption spectroscopy, following the absorbance of the oxidation product, ABTS^•+^, Figure 7B. Channels with grooves produced approximately twice the amount of product than in smooth-walled channels, for the same residence time (600 ms). This difference in conversion aligns well with analytical analysis, Figure 7B. In future studies, parameters such as electrode materials and scalability should also be considered in CFD studies. The difference in electron transfer rate associated with variation in electrode material and solute substrate can increase or decrease the relative importance of mixing quality.

### In-Line Analysis of Mixing and Flow Profiles in the Chip

**Figure 8:**
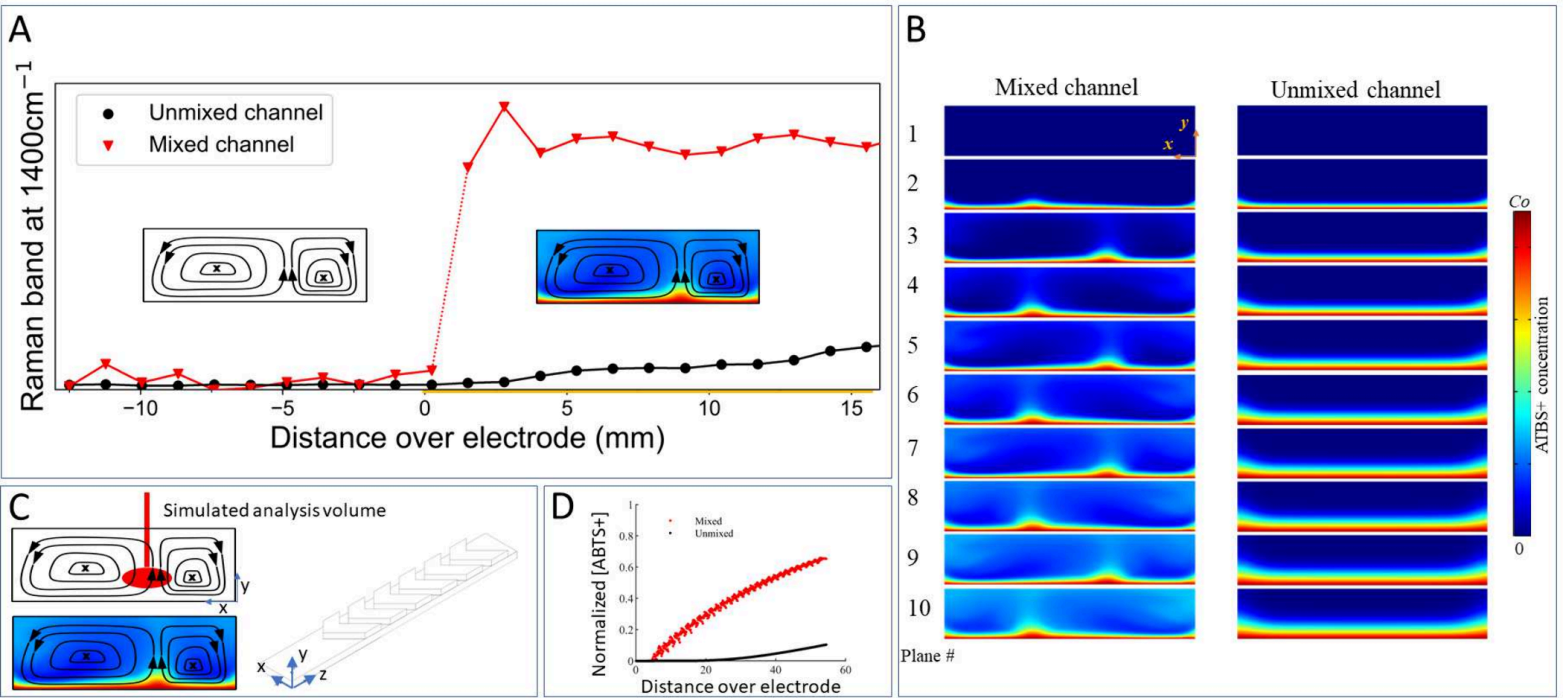
(A) Intensity of the Raman band at 1400 cm^–1^, measured at different axial distances on the electrode along a smooth and mixed microfluidic channel, respectively. This Raman band is associated with the oxidation product of ABTS, ABTS^•+^, which is produced in the channel. Therefore, the intensity corresponds to the amount of ABTS^•+^ produced. The production for the unmixed channel shows a slow increase, as is expected in laminar flow conditions due to the increasingly large diffusion layer. Measurements in the mixed channel feature a steeper increase in the ABTS^•+^ signal. This steeper increase is due to the confocal volume/sampling volume of the Raman measurement filling up with product. See Figure S4 for spectra of the products. (B) Simulated concentration profiles of ABTS^•+^ were obtained in the mixed and unmixed channels. The rows are axial cross sections along the length of the channel. The mixed channel features upwelling of product in the middle of the channel due to the convection. A reduction of the diffusion layer thickness is also observed, which indicates increased flux of reactant to the electrode surface. The unmixed channel features a diffusion layer that increases in thickness over time. (C) The volume probed by in-line Raman analysis is finite in size. The analysis volume is aimed at the electrode surface in the middle of the channel. This volume slowly fills up over the length of the channel for the unmixed channel but is engulfed by the upwelling of product in the mixed channel. (D) Simulated amount of product at the analysis location used in (A). A more rapid increase in product concentration is indeed expected.

The conversion achieved by inducing convection in the channels is increased substantially. However, understanding the interface structure and the impact of the grooves is essential to optimize the design for use in electrochemical processes. The difference in conversion between mixed and unmixed channels can be observed through Raman spectroscopic monitoring of changes at the interface, combined with CFD simulations. Here, the development of the Nernst diffusion layer is observed, Figure 8. An undisturbed diffusion layer particularly limits the currents achievable.

For the smooth-walled channel, a slight but gradual increase in product (ABTS^•+^) is observed by Raman spectroscopy along the length of the electrode, Figure 8A. This increase is consistent with expectations and simulations of diffusion layer behavior in Figure 8D. Indeed, the thickness of the diffusion layer should increase over time (or the distance under conditions of continuous flow). The applied flow limits the thickness of the diffusion layer at the start with fresh solution. However, over time and distance, the depleted volume will reach a steady state over the electrode surface. The confocal depth of a Raman microscope (ca. 5–10 microns using a standard 50× objective, Figure 8C) exceeds the Nernst diffusion layer established under continuous flow conditions. Therefore, the development of the diffusion layer in a channel without static mixing elements is readily observed experimentally by using this method.

The behavior for the channel containing mixers is more complex: a simple symmetric diffusion layer is not expected. Instead, the flow profile obtained by the grooves will draw product away from the electrode interface into the channel and away from the electrode. This flow behavior fills up the confocal volume of the Raman microscope more rapidly, which manifests in a substantial increase of product signal, as seen in Figure 8A. However, the rapid increase of signal cannot be directly correlated to an equivalent increase of electrochemical productivity when compared to the unmixed channel, because of the complexity of the flow profile.

Ultimately, the incorporation of grooves into the channel leads to mass transport becoming dominated by convection rather than diffusion (a change described by change in Peclet number) Specifically, a change in transverse Peclet number is observed with a higher transverse flow in mixed channels increases mass transport, whereas transverse flow in an unmixed channel is essentially zero;^24−28^ See SI for more information). Convection induced by the grooves increases the amount of fresh solute delivered to the electrode, which reduces the diffusion layer thickness. This increases the concentration gradient at the electrode and hence the current and conversion. Effectively, mass transport decreases the volume of the diffusion layer. In practice, the diffusion layer behavior is more complex; a symmetrical, constantly increasing boundary layer is not formed. Instead, non-axial fluid streams add to the complexity of the boundary layer shape expected for a mixed channel. Therefore, the finite confocal volume of a Raman microscope is unable to fully describe the diffusion layer. Simulations yield a clearer picture of the expected fluid behavior in the channel.

The expected shape of the boundary layer within the flow profile expected for the mixed channel was imaged from the simulated results for concentration, Figure 8B. Here, the drawing of product from the electrode interface becomes apparent: the double vortex produced by the mixers creates an upwelling of product on top of the electrode. Consequently, fresh solute is drawn in on the side of the channel to partake in new conversions.

The limits to the extents of achievable electrochemical conversions are of relevance in many applications. Although the fluid stream profiles are highly affected by the mixers, it should be noted that the mixers were not designed necessarily to ensure all solute in the solution reach the electrode interface (i.e., channel wall). For example, particles passing through the center of a vortex may move faster through the channel and exit without reaching the electrode at any point. Approaches to overcome this limit on conversion include using multiple devices in series or to oscillate the flow of the solvent back and forth in the chip, as the mixers equivalently push solvent to the electrode when flow is reversed, which leads to similar conversion efficiencies in both back and forward flow, Figure S5. These approaches ensure that a volume has more time and possibility to come in contact with an electrode. In future research, different designs to induce a more efficient convection of solute to the electrode can also be considered. Examples of future design considerations include a mirroring of the groove design (one channel with respect to the other) for more uniform pressures in the laminar flow channel and a Y junction instead of a T junction to reduce pressures at the start. Additionally, the geometry of the static mixer itself could be reconsidered, or optimized, to obtain the most efficient flux of solute to the electrode surface as well as different electrode materials such as glassy carbon.

### Simulation of ABTS Transport in SHM Domains

The fluidic interactions between SHM channels were evaluated by simulating ABTS transport through the entire device consisting of both left and right microchannels connected by a thin bridging section. In these simulations, ABTS flowed through only one inlet, where redox reactions with the electrode were not considered. The pressure at both outlets was set to zero. Numerical simulations demonstrated equivalent flow rates through each outlet (within 0.1%), which is also expected for experimental devices connected to outlet tubing that have similar fluidic resistance (i.e., same length and tubing diameter).

We observed a periodic steady-state oscillation of the ABTS distribution within the thin bridging channel along the axial direction (Figure S6a), which is also mirrored in the fluid streamlines (Figure S6b). This periodicity can be attributed to the non-symmetric design of the experimental device, where the mixing grooves are linear translations of one another (rather than being mirrored). Previous work has reported that non-axial flow within the long arm of each SHM groove will be higher than that within the short arm,^27^ which in the device considered here leads to a slight imbalance of pressure in the bridging channel (Figure S6c). This oscillatory behavior, however, does not lead to an appreciable level of solute crossover: at equal flow rates for both SHM inlets, the average concentrations of ABTS flowing through the right and left outlets were 0.998·*Co* and 0.0028·*Co*, respectively.

Further simulations were conducted to explore the effect of flow rate imbalance on the ABTS crossover, where we maintained a constant flow rate on the left SHM inlet while varying the flow rate of the right inlet. Figure S7a shows contours of the ABTS distribution throughout the inlet region for three different flow rates; as expected, channels that have a higher flow rate lead to filling the bridging region with the same solution in a relatively short axial distance. Due to the long length of the overall device with respect to the width of the bridging region, the viscous resistance of the bridging region has relatively no effect, and thus, the flow rate through both outlets is similar (<0.1%) for each simulation. The average ABTS concentration through each outlet is shown in Figure S7b. As expected, when the flow rate of the ABTS containing channel is higher than the channel with no solute, the concentration of ABTS through that outlet will approach that flowing through the respective inlet. These results demonstrate that reactant–product crossover can be mitigated by slight increases in flow rates through a channel of interest. For example, in the case where chemical reactions are occurring (e.g., ABTS is oxidized to ABTS^•+^ over the right electrode), the unwanted collection of side products (e.g., those being reduced in the opposite SHM arm) can be avoided by operating the device with a slight imbalance in flow rate.

### Simulations of ABTS Oxidation

We simulated ABTS transport through the SHM containing microchannels using the geometry and conditions specified in Table S1. We assumed fast electron transfer along the electrode surface (whose front edge was situated at a distance of 3 mm from the channel inlet) and that ABTS was converted into ABTS^•+^ with a 1:1 stoichiometry, where there were no other reactions of the two species on the electrode surface. To save computational memory, we only considered one arm of the experimental device by assuming that the device was symmetric in nature.

Figure 8B shows the simulation domain near the inlet region. We defined a series of planes situated between both full- and half-SHM cycles; at the axial distance of these planes, the fluidic domain is rectangular in shape (defined by *W* , the width, and *Hc*, the height of the channel), where the dynamics of ABTS depletion and ABTS^•+^ production are easier to visualize. The ABTS^•+^ profiles are plotted for both mixed and unmixed channels, the latter regarding flow in channels with a height of *Hc* and without SHM grooves. The contours for the mixed channels show distinct regions in which the ABTS^•+^ boundary layer has a localized increase in thickness, which is due to the local fluid uplift centered around the apex of the SHM groove. In the regions away from the groove apex, the boundary layers for the mixed case are thinner than those in the unmixed case (for a constant axial distance), which is followed by increases in ABTS^•+^ concentration in the upper regions of the channel.

The complex flow profile through the SHM thus leads to a complex pattern of localized ABTS oxidation on the electrode surface. These localized regions can be visualized via contours of the diffusive flux of ABTS normal to the electrode surface (Figure S6), which show large increases in flux (and thus rates of oxidation) near the edges of each groove pattern with respect to its apex. These are visualized in the figure as a red volume of the product emerging from the electrode surface (bottom) in the channel. In addition, periodic localized increases in flux (refreshment of solute) can be seen in the regions between grooves, where the channel height is smaller. Regions of decreased flux exist in the regions directly below each groove. Our results follow that of Kirtland et al.,^24,25^ which pertains to the diffusion-limited capture of material by a solid surface situated across from a SHM channel.

We probed these simulations to mirror the experimental Raman-based analysis of the channel cross sections; for each mixed and unmixed simulation, we defined a sphere that resembles the focal volume of the experimental setup (diameter 15 μm, offset by distances *x* and *y*), where for a set of axial (*z*) positions, we calculated the average ABTS^•+^ concentration within the sphere. It can be seen that the ABTS^•+^ concentration is much higher for the mixed channel, where the difference between mixed and unmixed results grows with an increasing distance from the electrode surface (*y*). At *x*-positions under the apex of the groove system, a periodic signal of concentration as a function of axial position can be observed, which is due to the changes in the localized uplift of the boundary layer seen in Figure 8B. Additionally, we observe here a significant difference in the concentration of product, as well as the difference between the mixed and unmixed channels, when compared to the unmixed channel. When measuring at positions away from the apex, this oscillatory behavior is much smaller.

### Applications

#### In-Flow Reductive Peroxide Generation on Pt Electrodes

An additional aspect of the transverse flow generated by the slanted herringbone grooves is its effect on the products of the electrochemical reactions. In laminar flow or batch electrochemistry, products generated at the electrode stay in the proximity of the electrode and can engage in further electrochemical reactions or interact with the electrode surface. When the product is short-lived or can react with the electrode surface, ideally, the product should be actively removed from the electrode solution interface. An example is the generation of H_2_O_2_ by reduction of O_2_ at a platinum electrode. Formed H_2_O_2_ can also undergo disproportionation to H_2_O and O_2_ at a platinum surface.^14^ However, the residence time in the channels of the current chip design is relatively short (<1 s). Therefore, the time that the product (e.g., H_2_O_2_) has to interact with the electrode again is also short. Hence, reactive products formed in the channel are quickly removed out of the channel.

**Figure 9:**
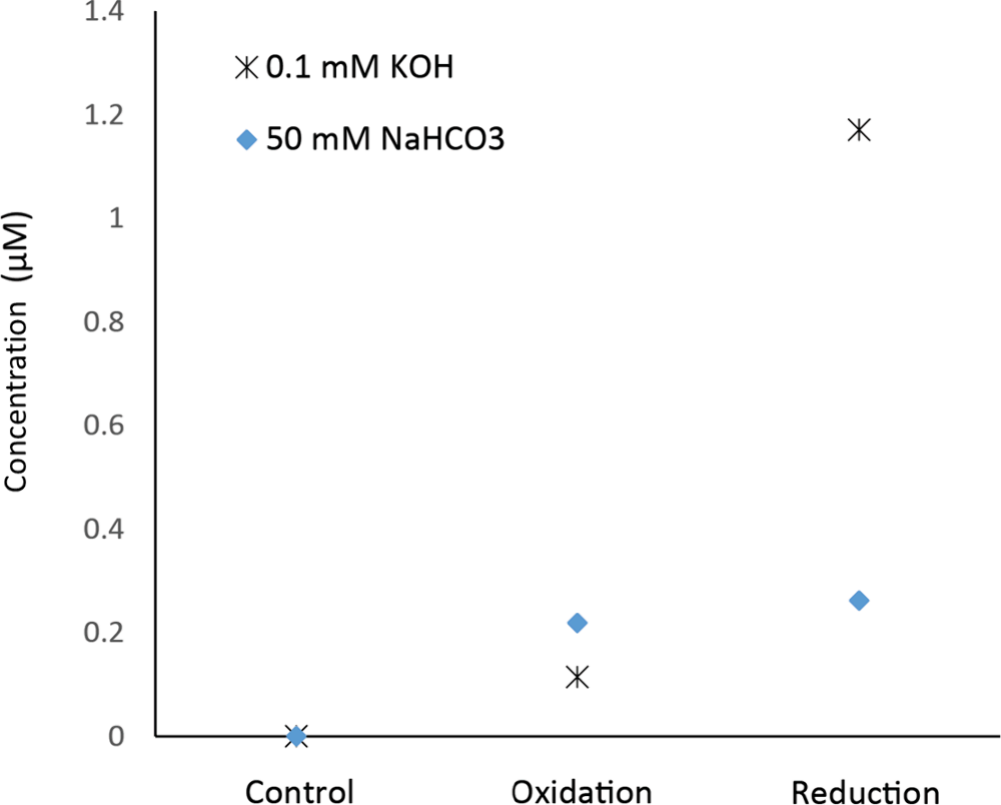
Quantification of hydrogen peroxide exiting the oxidative and reductive channels of the channel, under basic and acidic conditions, respectively, using platinum electrodes. For both experiments, identical solutions were pumped through both channels and the exits were analyzed separately. Platinum normally decomposes peroxide. Generated peroxide was quantified using the standard horseradish peroxidase method.^22^

Generation of H_2_O_2_ using platinum electrodes was carried out in the microfluidic chip as well as with bulk electrolysis using platinum mesh electrodes. H_2_O_2_ was not observed under bulk conditions as expected due to catalytic decomposition by the electrode. However, in flow with a high flow rate (100 μL/min, 600 ms residence time), H_2_O_2_ was detected under basic conditions. The hydrogen peroxide was generated in small amounts reductively from dissolved oxygen in the electrolyte solution, as shown in Figure 9. More mildly basic conditions, acidic conditions, and oxidation did not generate significant amounts of hydrogen peroxide.

#### Hydrogen Peroxide Generation at an FTO Anode

In situ H_2_O_2_ generation from O_2_ is potentially useful, and the current cell should be applicable to such an electrochemical reaction, Figure 9. H_2_O_2_ can be generated *in situ* to be used immediately by an (electro)catalyst. Besides platinum, there are more efficient ways to generate peroxide, e.g., from water using an FTO (fluorine doped tin oxide) electrode, Figure S8. This electrode material was used as well as a proof of concept chip that can produce H_2_O_2_, Figure S9.

#### Separation of Anode and Cathode Channels

**Figure 10:**
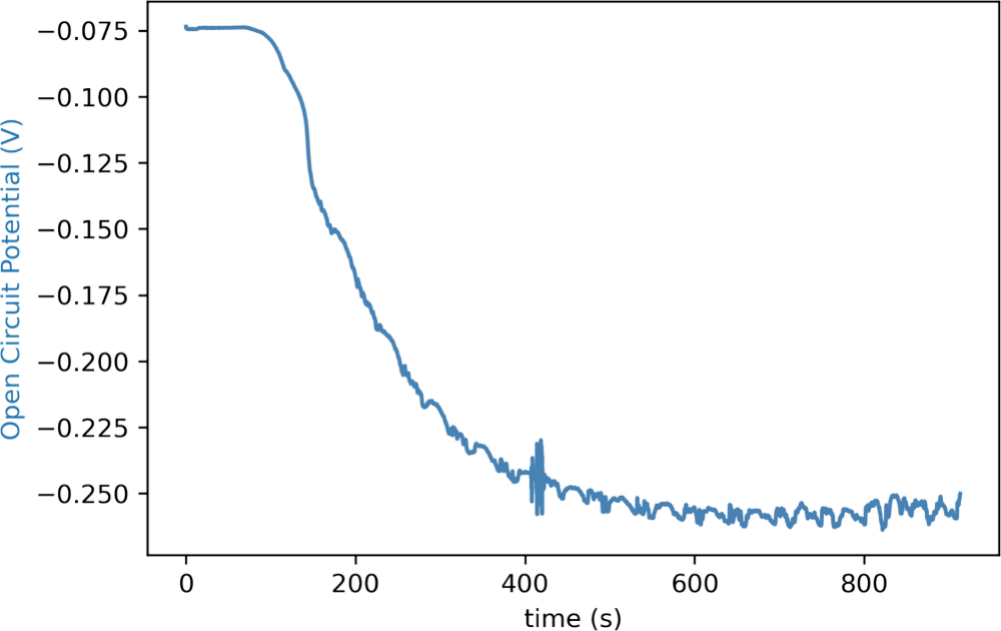
Open circuit potential over time due to a difference in pH between the solutions in each channel. A microreactor with gold electrodes was used. An aqueous solution of pH 4 and pH 10 was introduced in each of the channels at a flow rate of 0.11 mL/min each. Flow of the solutes equilibrated at 400 s.

The separation of the anode and cathode channels by a bridge that does not facilitate convective mass transport allows for oxidation and reduction in each channel to take place separately and remain separated. For example, an acidic and a basic solution entering the chip in separate channels remain separate over time. The separation is manifested in the measured potential difference, due to the difference in pH, between the channels, Figure 10.

## Conclusion

A microfluidic chip design for membrane-free divided cell electrochemistry is reported. The main features are long parallel electrodes to limit iR drop in separate anode and cathode channels. Channels are kept separate without a membrane via a bridge structure. Laminar flow through the shallow bridge channel prevents mass transport between the anode and cathode channels, but the solvent-filled bridge connects the channels electrically. Additionally, the mass transport is increased in both channels by static mixers. Mixing helps overcome diffusion limitations to a large extent by inducing transverse flow over the electrodes. This transverse flow thins the diffusion layer. As such, the addition of herringbone grooves as static mixers in the channels resulted in an increase in conversion of up to 100%.

The static mixer design increases overall electrochemical conversions, more than doubling efficiencies, though full conversion is not achieved with a single pass. The incomplete conversion is due to the relative higher axial velocity of the fluid in the center of the mixing vortices. Fluid passing through this passage will not come close enough to interact with the electrode and will therefore not undergo electrochemical conversion. Nevertheless, conversions of up to 55%, using a relatively high flow rate of 50 μL/min, can be achieved on a single pass. This limitation in conversion might be overcome using multiple devices in series or by oscillating the flow back and forth over the same device. While the single-pass conversion efficiency reached by the present approach is incomplete, Faradaic and power efficiencies are high due to the low iR drop between electrodes. As a result, the overall electrical and energy efficiency might be significant for devices similar to this in scaleup. Furthermore, the overpotential required is low as a result. Additionally, the efficiency per electrode area is limited only by mass transport and not iR drop.

In principle, we expect the device to be broadly applicable due to the variation in solvents, pH, conditions, and electrodes that can be employed in the chip. Electrode materials are more challenging to change but this is still relatively straightforward with the rapid prototyping approach as presented here.

We anticipate that the current chip design will find use in the operando analysis of electrochemical or electrocatalytic reactions. In particular, in situ generation of hydrogen peroxide can be useful for kinetic analysis of electrocatalytic reactions, and steady-state flow can be used to investigate formed electrochemical species with high time resolution.

## Experimental Section

### Materials

Solvents (spectrophotometric grade) and reagents were obtained from commercial sources and used as received, unless stated otherwise. The silicone rubber polydimethylsiloxane (PDMS, Sylgard 184) was obtained from, Mavom BV, Alphen a/d Rijn, The Netherlands.

### Fabrication of Microfludic Electrochemical Chips

The microfluidic chip was fabricated in two parts: the 3D part containing the channels and the flat glass slide on which the electrodes were deposited. The chip design employed here contains two main channels for the anode and cathode, with slanted herringbone groove structures on the ceiling to promote mixing. These herringbone grooves were chosen due to the large dynamic range of flow rates that they work under and their effective mixing rates.^21,28^ The two channels are connected along the entire length via a 10 mm wide channel. The bridging section does not contain static mixers, and therefore the flow is purely laminar and axial. These channels are fabricated using PDMS, which adheres well to the glass base. Adhesion to the areas covered by the electrode was less good. Therefore, the electrode design was modified to ensure good overall bonding of the PDMS and glass components.

Microfluidic channels were prepared by using standard lift-off photolithography methods. A master (3D mold) was prepared using EPON SU 8 photoresists (Micro resist technology GmbH, Berlin, Germany) with a mask drawn in the software package CleWin32, printed by Pro Art BV, Groningen, The Netherlands. The mask has dark areas to block UV light selectively in the shape of the desired microchannel geometry. The resulting resist structure can be selectively developed and hardened onto a glass wafer. The current design was built using three of these photoresist layers stacked on one another. When fully cured, the wafer containing the desired channel geometry was used as a mold over which liquid PDMS prepolymer was poured. The mold, with prepolymer on top, was then heated to 70 °C to form and cure the PDMS. The cured PDMS was peeled off yielding the desired pattern of the channels.

The channel geometry and herringbone groove structure/pattern were based on CFD (computational fluid dynamics) optimizations reported earlier, Figure 7.^28^ The geometry consists of a base layer, which includes both the main channels and the thin, laminar flow region, or bridge, between the channels. This layer is followed by a second channel layer, which forms the main body of the channel volume. The last top layer contains grooves that act as static mixers. A second geometry, in which the grooves were omitted, was employed as a control “unmixed” chip. The height of this unmixed channel was chosen such that the flow speed at the electrodes would be equivalent to that in the channel containing groove structures at the same flow rates.

Electrodes were deposited on the glass base of the microfluidic device using physical vapor deposition (Kurt J. Lesker, NanoLab-NL, Groningen, Netherlands), covering a significant length of each channel (4.5 cm). The electrode pattern was formed using a steel mask and fabricated in-house using wire electrical discharge machining, Figure S10. A titanium adhesion layer (5 nm) was deposited on the glass, followed by sputtering of the electrode material to a thickness of 100 nm at a rate of 0.77 nm/s. Gold and platinum electrodes were used in most experiments; however, iron and nickel also produced working devices, Figure S11.

In addition, a device was prepared using fluorinated tin oxide (FTO) coated glass (Sigma-Aldrich) for the generation of H_2_O_2_.^29^ A scratch of approximately 300 micrometers in width was made through the FTO layer to separate the slide into two electrodes (forming the anode and cathode in the device). Platinum was sputtered on one side of the scratch as a counter electrode, Figure S12.

### Voltammetry

Voltammetry was carried out using a CHI604e potentiostat with connections made via one of the three connector pads on each side of the device. Three pads for connections are included, as the nanometer thick electrode can wear, which would render the chip useless. A piece of platinum mesh was placed between the electrode and crocodile clip to ensure reliable contact, Figure S13. It should be noted that without the platinum mesh, significant variation in resistance of several orders of magnitude of the devices is measured.

Electrolytes containing chloride were avoided specifically due to possible anodic stripping of gold from electrodes. The counter electrode, in water, is essentially a standard hydrogen reduction electrode (normal hydrogen electrode, NHE), which allows for potentials to be applied relatively accurately.

Throughout the current contribution, water is used as a solvent, containing a 0.1 M electrolyte (KNO_3_). In these conditions, water is an excellent counter reaction, producing hydrogen reductively and oxygen oxidatively, at relatively low potentials. We used water electrolysis as counter-reaction for all experiments.

### In-Line Analysis

The performance of the electrochemical chips was evaluated by in-line Raman microspectroscopy at λ_exc_785 nm (65 mW at source, Ondax LMR-785, CA, USA). Spectra were collected by using a backscattering optical configuration. A dichroic beamsplitter (Semrock Di02-R785-25) was used to bring the excitation beam, colinear with the optical axis of the spectrometer, to the sample through an Olympus 10× objective. In this path, laser intensity was controlled using a λ/2 retarder and a polarizing beamsplitter. The beam diameter was expanded to increase the *z*-confocality of the system to ensure that the confocal volume of the microscope matched the channel depth. Raman scattering was collected in 180° backscattering mode and passed through a Rayleigh rejection filter (long-pass filter, Semrock LP02-785RE-25) before it was focused with a 2.5 cm diameter (3 cm focal length) planoconvex lens into a Shamrock193i spectrograph. The spectrograph was equipped with an iDus-416 CCD camera (Andor Technology) as well as a 1000 l/mm grating blazed at 780 nm. Spectra were calibrated with the spectrum of polystyrene (ASTM E1840-96 Standards for Raman spectroscopy, 2002). Spectra were analyzed by using SpectraGryph-12 and plotted using Python.

Analysis using in-line UV/vis absorption spectroscopy took advantage of the good transmission of both the glass base and the PDMS materials of the device, Figure S14. A fiber-coupled spectrometer (AVASPEC-ULS2048CL-EVO, Avantes, the Netherlands) and a fiber coupled halogen light source were used to record spectra in situ, Figure S15. Electrolyte was pumped through the channel when potential was applied during in-line measurements.

### Flow Control

Solutions were pumped through the channels using syringe pumps (New Era Syringe Pumps, New York, USA) and 5 mL syringes. Blunt needles were attached to the syringes and were fitted snugly in 1 mm diameter PTFE (polytetrafluorinated ethylene, Polyfluor Plastics b.v., Breda, The Netherlands) tubing. For the entrance on the chip, an identical blunt needle was used, stripped of its syringe connector. One end of the needle was glued inside the chip during PDMS curing (70° C in an oven for 30 min). The other end of the needle, Figure 5D, was connected to the syringe via the PTFE tubing. Tubing, needles, syringes, and the chip were filled with liquid before each experiment to avoid bubble formation. Pumps on in- and outlets were leveled at the same height to avoid pressure gradients across the chip. Aspirating pumps were always turned on first to prevent overpressure and leaks. Small leaks, e.g., at needles, were plugged using clear epoxy.

### Determination of Uncompensated Resistance (iR Drop)

The uniform potential along the length of the electrode was verified using a stopped-flow experiment; the channels were filled with a solution of ABTS (0.6 mM) in an aqueous electrolyte solution (0.1 M KPF_6_). After the flow of solution was stopped, a potential of 1 V was applied briefly across the cell to induce oxidation of ABTS to ABTS^•+^. The amount of ABTS^•^ generated along the electrode held at positive potential (anode) was determined by in-line Raman spectroscopy, taking advantage of the resonance enhancement of the Raman scattering of ABTS^•+^ at 785 nm.^21^ Raman intensities of ABTS^•+^ at varying locations along the channel can be compared to investigate the rate of conversion. The conversion to ABTS^•+^ was found to be constant along the length of the channel, confirming a constant iR drop.

### Finite Element Simulations of Fluid Flow and Reactant/Product Transport

Numerical simulations were carried out using the finite element solver, COMSOL. The simulation domain consisted of an inlet channel delivering reactant to a main channel having staggered herringbone mixer (SHM) grooves, the bottom of which consisted of an electrode serving to oxidize the reactant. We assumed the electron transfer process was much faster than the transport of reactant to the electrode (i.e., diffusion-limited) and, furthermore, that this process was irreversible (i.e., sufficiently large overpotential is applied). Steady solutions of the Navier-Stokes equations were used to obtain solutions of the convection-diffusion equation for both the reactant (concentration C1) and product (concentration C2), where an irreversible surface reaction was applied as a surface boundary condition on each electrode surface (C1 to C2). All solutions were solved as steady state, where solutions for flow and diffusion were obtained using a multigrid method using successive over-relaxation for both the pre- and postsmoother, with the PARADISO method for the course solver. We instituted three iterations for both flow and diffusion (the latter of which used quadratic elements), both having relative tolerance criteria of 0.001. Mesh size is an important parameter in finite element simulations. We evaluated the dependence of the mesh size on the solution outputs for the simulations below, Figure S6 and Table S1.

## Data Availability

The data that support the findings of this study are available in the Supporting Information of this article, and primary data is available on request to the authors.

## Supporting Information Available

Experimental methods, discussion of CFD calculation methodology, and additional data and spectra (PDF)
