# Supplementary material for: Electrochemical Flow Reactors: Mass Transport, iR Drop, and Membrane-Free Performance with In-Line Analysis
Source: ACS Electrochem. 2025 Jan 14;1(4):504–15. doi: 10.1021/acselectrochem.4c00167 (PMC11973871; doi:10.1021/acselectrochem.4c00167)
Supplement: Supplementary file 1 — ec4c00167_si_001.pdf [file ec4c00167_si_001.pdf]

Supporting information

Electrochemical Flow Reactors: Mass Transport, iR Drop, and Membrane-Free Performance with In-Line Analysis

W.J. Niels Klement,[a] Elia Savino,[a] Sarah Rooijmans,[a] Patty P. M. F. A. Mulder,[b] N. Scott Lynn Jr.,[*,c] Wesley R. Browne[*,a] and Elisabeth Verpoorte,[*,b]

[a]Molecular Inorganic Chemistry,Stratingh Institute for Chemistry, Faculty of Science and Engineering, University of Groningen, Nijenborgh 3, 9474AG Groningen, the Netherlands

[b]Pharmaceutical Analysis, Groningen Research Institute of Pharmacy, University of Groningen, Antonius Deusinglaan 1, 9713 AV, Groningen, The Netherlands

[c]Institute of Physics of the Czech Academy of Sciences, Na Slovance 1999/2, 18200, Czechia

email: lynn@fzu.cz, w.r.browne@rug.nl, e.m.j.verpoorte@rug.nl

	Page
Additional figures	S1
CFD mesh size test	S10
Analytical solution for chemical transport in SHM channels	S11

Additional figures

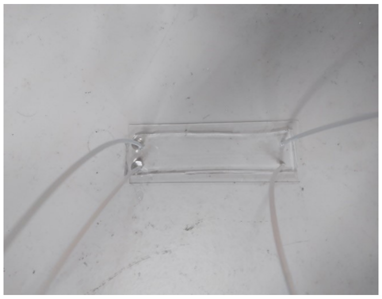

Figure S1: Electrode free analogue of the chip with blue liquid flowing through the top channel, and water flowing through the bottom.

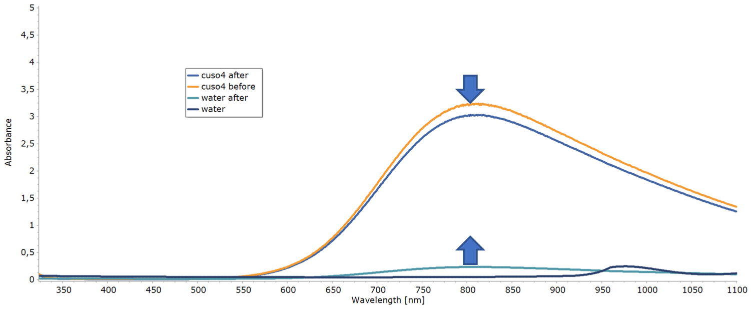

Figure S2: UV/vis absorption spectra of channel separation measurement. Copper sulfate solution flowing into one side of the device was slightly diluted at the outlet, whereas the water from the other side had become slightly colored from the outlet. see Figure S3 for photo of solutions

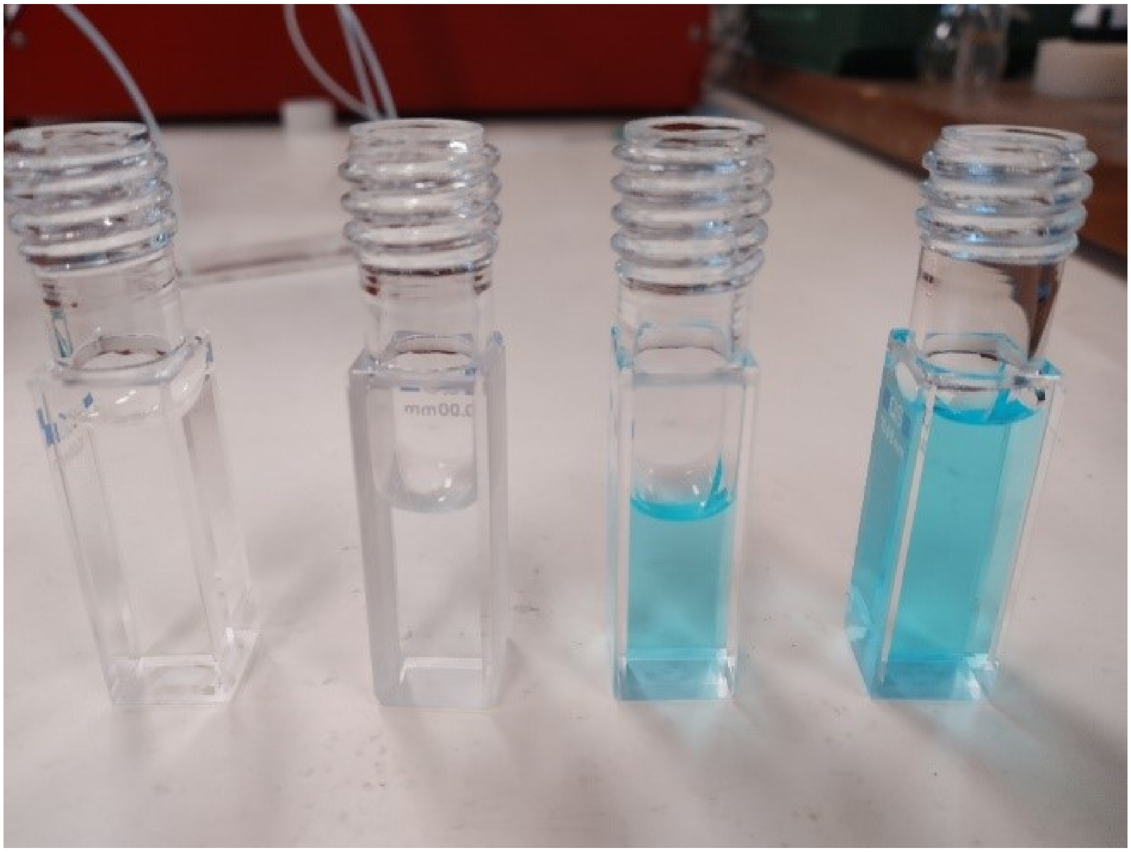

Figure S3: Solutions flowing in (outer cuvettes) and out of chip (center cuvettes) in FigureS1. The essentially colorless liquid emerging from the non-colored channel (the center left cuvette) indicates that the migration of species is low.

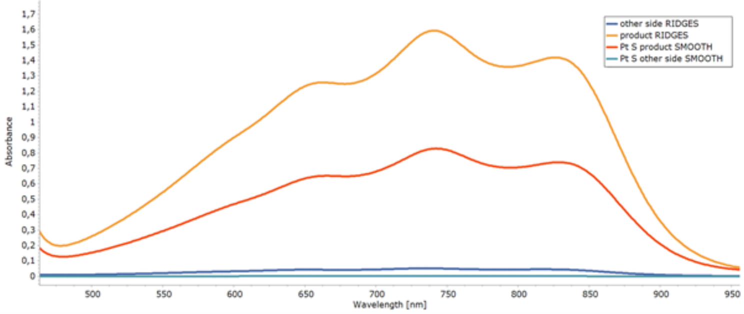

Figure S4: UV/vis absorption spectra of $ABTS^{\cdot+}$ in water, produced from 0.65 mM ABTS in water on an electrochemical microfluidic device with (yellow) and without (red) herringbone mixers. Channels included platinum working and counter electrodes. Potential difference is 1.1 V and flow rate 100 μl/min. The exact amount of crossover was determined to be 5% with UV/vis absorption, Figure S2. Since the residence time is too short to cause migration of species at this quantity, convective effects from fluids entering the device and meeting in the center are expected to be the cause of this exchange of fluid between the two sides of the device.

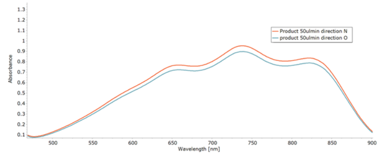

Figure S5: Absorption spectra of ABTS, oxidized in the microfluidic device while flowing normally (N) or in the other direction (O). The similar conversion indicates that backwards flow would yield similar efficiencies, which could enable different operation of the device, such as oscillated flow, to increase the available conversion.

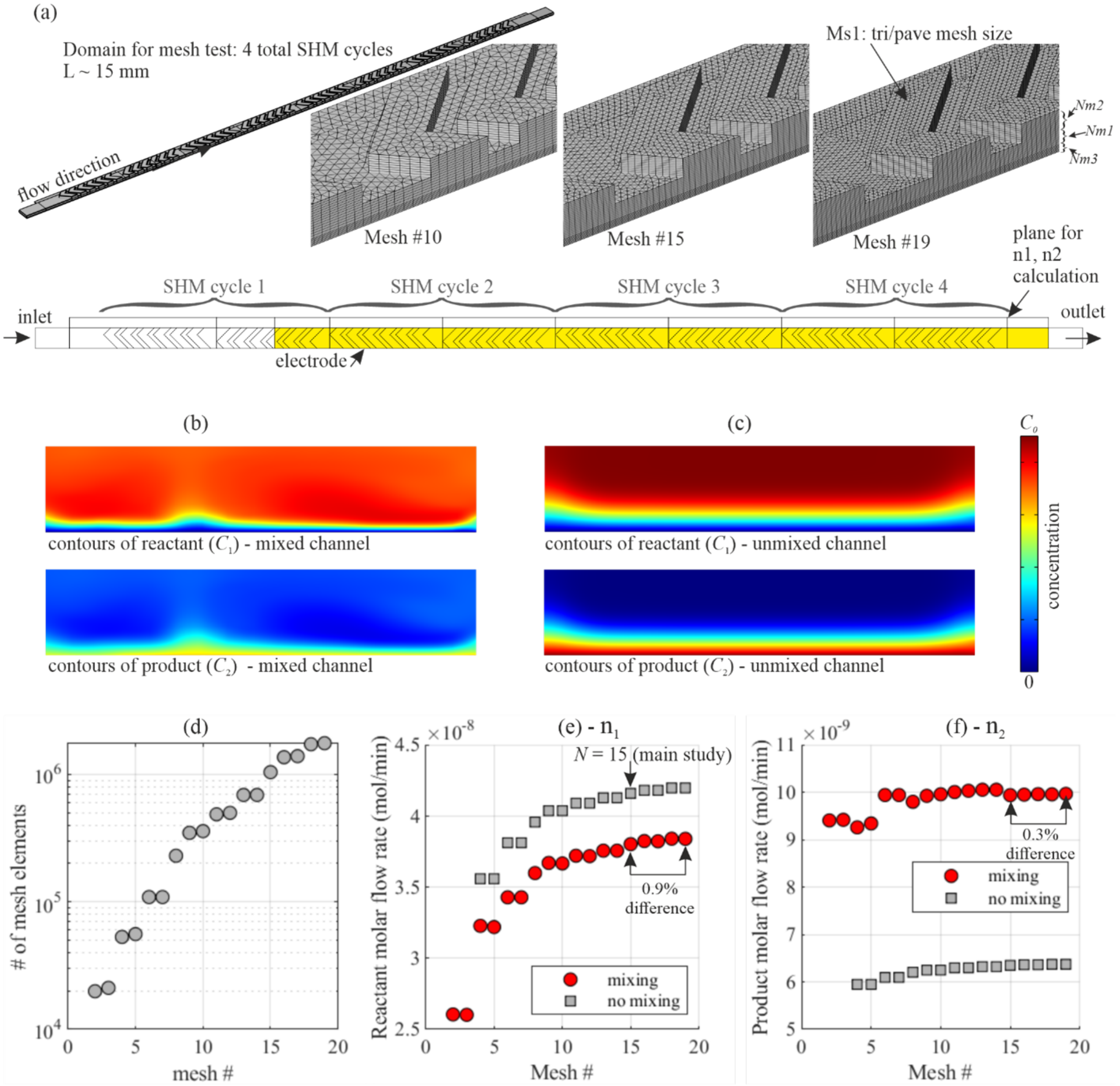

Figure S6: (a) Computational domain used in this study along with zoomed in regions of the mesh at values of N = 10,15, and 20. (b,c) Contours of reactant and product concentration along the calculation plane (situated downstream of the 4th SHM cycle) for both mixed and unmixed geometries, used for the calculation of both n1 and n2. (d) Total number of meshing elements vs. the mesh # for mixed simulations. (e,f) Molar flow rate of the reactant and product vs. the mesh #, both of which were calculated from the contours similar to that shown in (b) and (c).

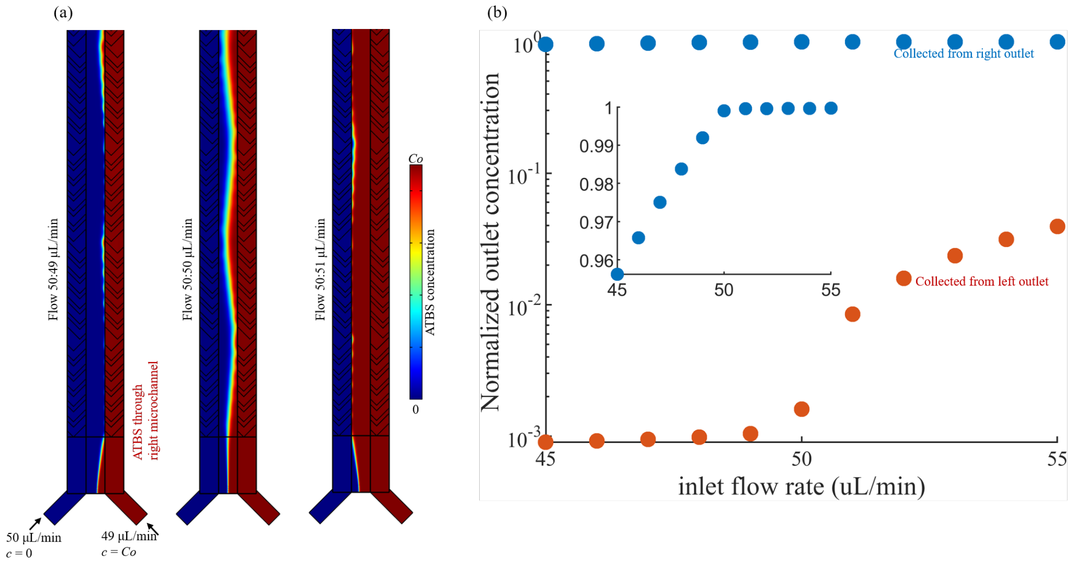

Figure S7: (a) Computed [ABTS] along channels as a function of differences in flow rates. (b) Simulation of transverse flow between anode and cathode channels for various flow rates.

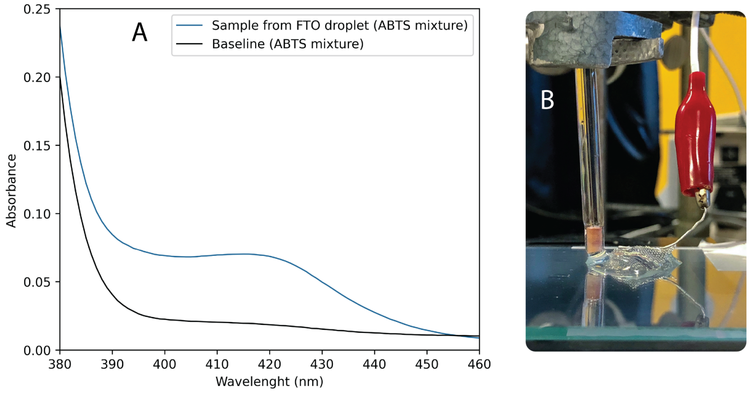

Figure S8: A) UV/vis absorption spectra of an ABTS assay, containing one control sample (without hydrogen peroxide), and a sample taken from the droplet after 2 h of continuous electrolysis at 1.4 V. A clear absorbance compared to the baseline is observed, indicating hydrogen peroxide formation. B) photograph of the setup employed for the FTO experiment.

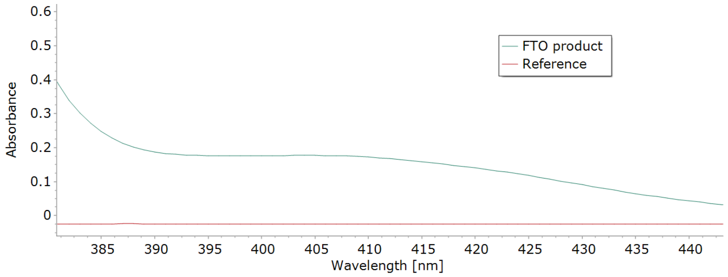

Figure S9: UV/vis absorption spectra of an ABTS assay, containing one control sample (without hydrogen peroxide), and a sample taken from the anode channel of the chip after electrolysis at 1.4 V using a flow rate of 50 μL/min. The anode electrode was FTO and the cathode platinum.

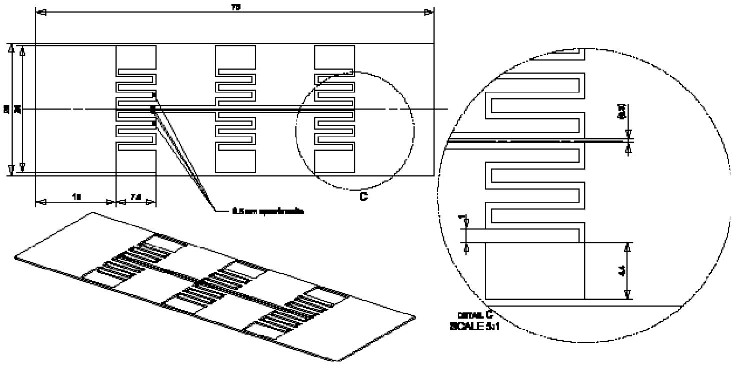

Figure S10: Mask used in fabrication of electrodes on glass. Patterns were cut in stainless steel (0.3 mm thick) using wire EDM

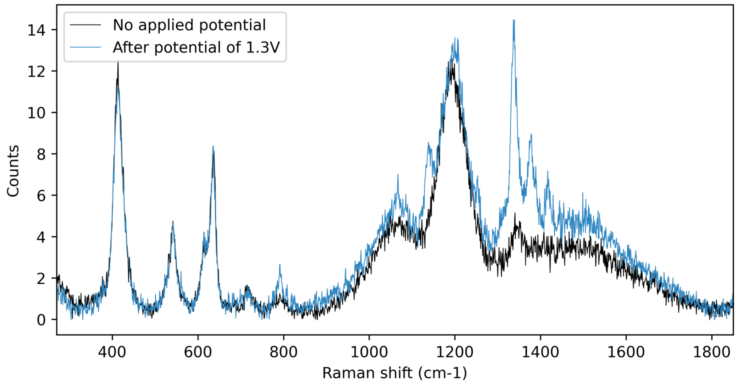

Figure S11: Raman spectra of ABTS in an Ni/Fe flow chip, before and after a potential was applied showing the appearance of the $ABTS^{\cdot +}$.

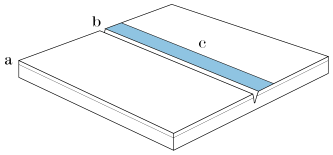

Figure S12: FTO slide a) FTO coating on glass. b) cavity formed using a glass scoring tool. This cavity electrically disconnects the top coating into to areas . c) a platinum strip deposited on top of the FTO electrode on one side of the score.

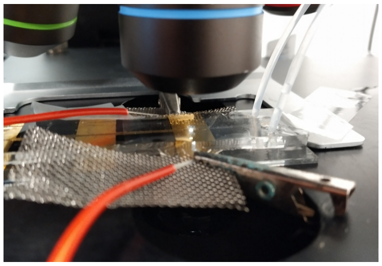

Figure S13: Platinum mesh used to increase the conductivity of connections between the potentiostat and the electrodes of the channel.

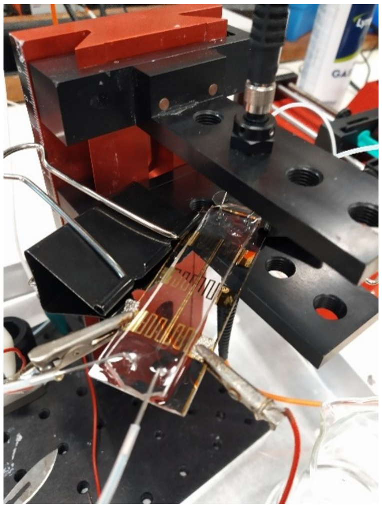

Figure S14: Arrangement for spectroelectrochemical in-flow measurements of changes in UV/vis absorption in a microchannel on the electrochemical chip. Orange optical fibers guide the light from the source, via the chip to the spectrometer. Syringe pumps regulate flow and the potentiostat controls applied electrical potentials. Electrodes are attached to the potentiostat via crocodile clips and the illuminated spot at the end of the channel is the on-chip detection location.

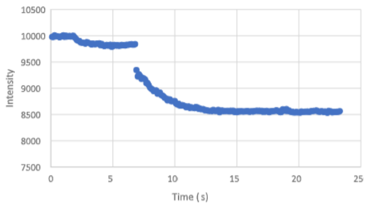

Figure S15: Change to transmitted light at 420 when a potential (0.6 V) is applied across the chip. A decrease in transmitted light is observed, due to absorption by $[Fe(III)(CN)_6]^{3-}$ at 420 nm.

CFD mesh size test

We evaluated the dependence of the mesh on the solution outputs. For purposes of computational complexity these mesh convergence simulations were performed on a shortened fluidic domain consisting of 4 full SHM cycles (experimental device uses 16). Furthermore, only one fluidic channel is considered, where we assumed that reactant and product transport across the bridging channel was minimal and thus imposed a symmetry condition along the center of the full device. For this, the fluidic domain was meshed in a procedural fashion as a function of a mesh # (N, Table S1), which resulted in a mesh size MS1 in the horizontal plane (x,z), and was applied to a tri/pave scheme. The vertical edges of the main portion of each channel was split into *Nm1* elements, where the groove had *Nm2* vertical elements, and the lower boundary layer section (including the bridging channel) was split into two regions, each having *Nm3* vertical elements. The tri/pave mesh was then swept through each domain to create a series of triangular prisms. For all simulations the molar flow of reactant was calculated as $n1 = \int\int vC_1 dA$, the product as $n2 = \int\int vC_1 dA$, where v is the overall fluid velocity and the integrations were performed over a plane orthogonal to the flow axis situated at the end of the last SHM cycle (Figure S6b,c).

The results of the mesh convergence test are shown in Figure S6; these simulations were initialized with the parameters shown in Table S1. As expected, the molar flow of reactant (n_1) is lower for the SHM-mixed channels when compared to an unmixed channel for all mesh densities; in a similar fashion the concentration of product (n_2) is higher in mixed channels. The molar flow rate of the reactant (Figure S61e) is more sensitive to the mesh # with respect to the product (Figure S6f), as the latter is primarily present in the lower (boundary layer) portion of the channel where there are more meshing elements. Both n_1 and n_2 display convergent behavior at high mesh densities. A mesh # of $N = 15$ was chosen for the main simulations in this study (1.0M elements for 4 SHM cycles): simulations at higher mesh density ($N = 19$, 1.8M elements) give a solution of both n_1 and n_2 that deviate by less than 0.9% and 0.3%, respectively. For simulations of 16 SHM cycles, a mesh # of N

= 15 corresponds to a domain with 3.9M elements, which requires approximately 75GB of computational memory and $\tilde{4}$ h to solve.

Description	Parameter	Value
geometrical parameters		
Main channel height	*Hc*	60 μm
Inlet channel width	*W*	300 μm
Bridging channel height	*Hb*	10 μm
Axial length of groove	*lg*	156 μm
Axial distance between grooves	*Λ*	242 μm
# of grooves per ½ cycle	*Ng*	6
Volumetric flow rate (each inlet)	*Q*	50 μL/min
Diffusivity of reactant/product	*D*	4.4×10^{-4} mm^2/s
Inlet concentration of reactant	*Co*	1 mM
meshing parameters		
mesh number	*N*	
horizontal mesh size (tri/pave)	*Ms1* = 3(*Hc*-*Hb*)/*Nm1*	
# of vertical elements in main channel	*Nm1* = ceil((*Hc*-2*Hb*)/(*Hc*+*Hb*)·*N*)	
# of vertical elements in boundary layer	*Nm2* = 1+ceil((*Hb*)/(*Hc*+*Hb*)·*N*)	
# of vertical elements in groove layer	*Nm3* = ceil((*Hg*)/(*Hc*+*Hb*)·*N*)	

Table S1: Parameters used in mesh test

Analytical solution for chemical transport in SHM channels

. The problem of heterogeneous diffusion-limited solute transport to a reactive boundary in chaotically mixed channels has been analyzed in detail by Kirtland et al.[24] and confirmed experimentally in SHM channels in a later study.[26] These studies have shown that solute transport to a solid boundary must be examined from a local perspective, mainly through $j_z = j_z(z)$: the normal diffusive solute flux to the electrode surface averaged across the width of the electrode at an axial distance z from the start of the electrode surface. This value can be used to define a local Sherwood number $\mathrm{Sh_z}$, a dimensionless value that estimates the efficiency of solute transport, which can be calculated as

$$Sh_z(z) = \frac{j_z H_c}{C_b D} \quad eq.S1 \tag{1}$$

where $C_b = C_b(z)$ is the mixing-cup solute concentration, which can be defined as

$$C_b = \frac{1}{Q} \iint c(x,y) v_z(x,y) dxdy \quad eq.S2 \tag{2}$$

where v_z is the fluid velocity in the axial direction, and c is the local solute concentration throughout the SHM channels. This mixing-cup concentration can be simply thought of as the concentration one would obtain by collecting the flow through the channel (at a given distance z), and thoroughly mixing the fluid in a small cup. Another useful parameter here is the inverse Graetz number $\bar{z}$, a dimensionless number describing how the solute boundary layer behaves in an unmixed system, calculated here as

$$\bar{z} = \frac{zD}{QWH_c} \quad eq.S3 \tag{3}$$

For *unmixed* channels like those in this study, both Sh_z and $\bar{z}$ describe the behavior of solute transport under all conditions (*e.g.,* variable flow rate, solute diffusivity, channel dimensions, *etc.*), where these parameters will exhibit the following trends:

- In the entrance region, defined as $\bar{z} << 1$, solute boundary layers will remain small with respect to the channel height, and the local Sherwood number will scale as $Sh_z \propto \bar{z}^{-1/3}$.

-In the fully developed region, defined as $\bar{z} > 0.1$, solute boundary layers will have reached the upper surface, and the local Sherwood number will tend toward an asymptotic value of $Sh_\infty = 2.45$.

For *chaotically mixed* channels (exhibited by the SHM), the local Sherwood number will tend towards a higher asymptotic value of $Sh_\infty \geq 2.45$, where for fluids flowing sufficiently fast (where transport via convection is much higher than transport via diffusion, or simply, flows with a large Péclet number), that asymptotic value can be approximated by

$$Sh_\infty \approx \frac{U_t H_c}{D} \quad eq.S4 \tag{4}$$

where here, U_t is the average non-axial flow (measured in the x-direction) induced by the mixing grooves. For both unmixed and chaotically mixed fluids, the local Sherwood number can be estimated as

$$Sh_z = (\bar{z}^{-5/3} + Sh_\infty^5) \quad eq.S5 \tag{5}$$

Figure S16a plots this equation against computational results obtained in a mixed channel, where it can be seen that increases in Sh_∞(i.e., chaotically mixed channels) lead to deviation from that observed in an unmixed channel. It must be stressed that the results shown in Figure S16a cannot be directly tied to results from experiment, that is, the flux of solute to an electrode (directly related to the produced current) is not proportional to changes in Sh_∞, nor changes in Sh_z. Nevertheless, the mixing-cup concentration throughout an experimental device can be calculated as

$$C_b(z) = C_o exp\left(-\int_0^z \frac{Sh_z D}{QWH_c} dz\right) \quad eq.S6 \tag{6}$$

where Sh_z is calculated directly from Eq. (5), which requires only an estimation of Sh_∞ in an experimental device. If desired, one can then calculate the local flux from both Eq. (5) and (6) as

$$j_z(z) = \frac{Sh_z D C_b}{H_c} \quad eq.S7 \tag{7}$$

where it follows that the average values of j_z along the entire length of the electrode can then be used to estimate the current produced by the oxidation of **ABTS**. Figure S16b shows comparison of results obtained from numerical simulation (via Comsol with the mesh shown above) and those derived using Eqs. (5) and (6).

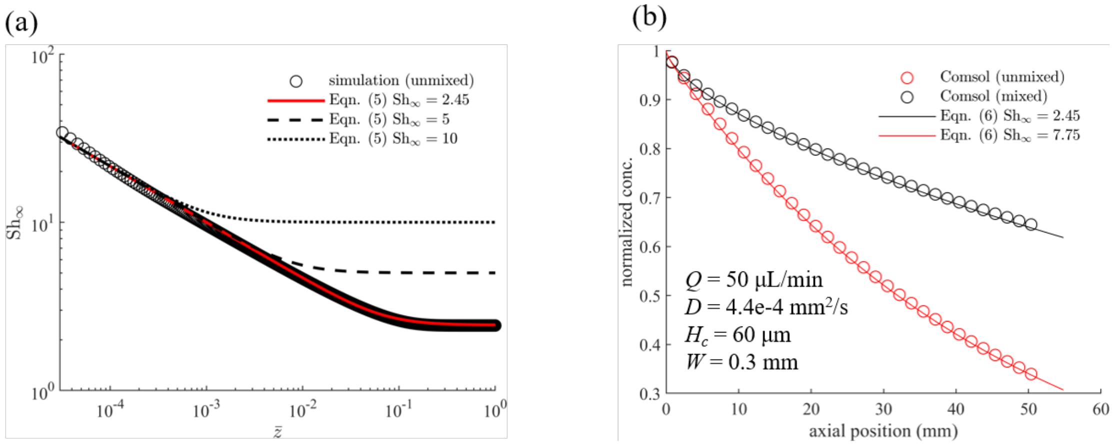

Figure S16: (a) Plots of result of equation S5 vs computational results obtained in a mixed channel. (b) Comparison of results obtained from numerical simulation (via Comsol with the mesh shown above) and those derived using Eqs. (5) and (6)
